# Dietary Habits and Their Correlation with Socio-Demographic Variables Among the Ethnic Hungarian Population of Romania

**DOI:** 10.3390/nu17050756

**Published:** 2025-02-21

**Authors:** Francisc-Andrei Boda, Béla Kovács, Bernadett Molnar, Boglárka Kovács-Deák, Lavinia Berța

**Affiliations:** 1 Department F1, General and Inorganic Chemistry, George Emil Palade University of Medicine, Pharmacy, Science and Technology of Târgu Mures, 540142 Târgu Mures, Romania; francisc.boda@umfst.ro (F.-A.B.); lavinia.berta@umfst.ro (L.B.); 2 Department F1, Biochemistry and Environmental Chemistry, George Emil Palade University of Medicine, Pharmacy, Science and Technology of Târgu Mures, 540142 Târgu Mures, Romania; 3 Faculty of Medicine, George Emil Palade University of Medicine, Pharmacy, Science and Technology of Târgu Mures, 540142 Târgu Mures, Romania; molnar_bernadett@yahoo.com; 4 The Doctoral School of Medicine and Pharmacy, Institution Organizing University Doctoral Studies, George Emil Palade University of Medicine, Pharmacy, Science and Technology of Târgu Mures, 540142 Târgu Mures, Romania; bogyo131@gmail.com

**Keywords:** consumer preferences, dietary patterns, ethnic minorities, food choice, healthy diet, nutritional habits

## Abstract

**Background/Objectives**: Non-communicable diseases (NCDs) are the leading cause of preventable morbidity and mortality globally. To reduce the prevalence of NCDs, the World Health Organization issued guidelines for a healthy lifestyle, which have been adopted in various countries. Our study aimed to evaluate the dietary habits of the ethnic Hungarian population of Romania, allowing us to identify potential differences in nutritional behavior compared to the country’s general population. **Methods**: A cross-sectional, observational, questionnaire-based study was conducted to collect information on eating behavior, food purchasing habits, and dietary patterns among ethnic Hungarians, the largest minority group in Romania. The obtained data were interpreted using multivariate data analysis (MVDA), including principal component analysis models (PCA-X) to establish pattern recognition and data clustering, and orthogonal partial least squares discriminant analysis (OPLS-DA) models to examine class differences between the identified clusters. **Results**: A total of 247 valid questionnaires were evaluated; the most represented groups were females (67.2%), young adults aged 18 to 30 (56.3%), individuals with normal body mass index (54.7%), and those with a higher education level (45.7%). Health-conscious purchasing and eating behaviors were more characteristic of middle-aged and older adults, females, and those with a higher education level. Young adults appear to have a more varied diet, but overconsumption of unhealthy food products and a lack of interest in healthy dietary habits is evident. **Conclusions**: Appropriate nutritional education is necessary for all age groups; however, programs targeting young adult Hungarians are especially important, as most expressed little interest in healthy eating habits. Further research examining the underlying relationship between dietary habits and cultural factors as well as socio-economic factors could offer new opportunities to promote a healthy lifestyle.

## 1. Introduction

Non-communicable diseases (NCDs) are defined as chronic conditions that are not caused by pathogens but develop over a long period due to behavioral, biological, or environmental factors. NCDs are the leading cause of preventable morbidity and mortality worldwide, with the latest progress monitor released by the World Health Organization (WHO) highlighting that NCDs are responsible for 74% of deaths worldwide [1,2,3]. Furthermore, these numbers continue to increase, as the highest risk of premature death caused by NCDs is in low- and middle- income, developing countries, which account for more than three-quarters of the global population [4,5]. In an effort to reduce the incidence of NCDs, the WHO issued guidelines with recommended measures at a global, national, and individual level.

The Joint Expert Report released by the World Health Organization (WHO) and the Food and Agriculture Organization (FAO) of the United Nations states that the two main pillars of a healthy lifestyle, physical activity and healthy diets, should be recommended and promoted to the general population. Proposed key elements of healthy dietary habits include a mainly plant-based, varied diet to satisfy the energy, protein, vitamin, and mineral requirements of the body. Additionally, the energy intake should be aligned with its consumption, with total fats less being than 30% (mainly as unsaturated fats) and refined sugar being less than 10% of the total energy intake. Legumes and wholegrain cereals are proposed as the main source of carbohydrates, supplemented with the daily consumption of vegetables and fruits, for both adults and children. The report proposes that healthy dietary habits should be complemented by an active lifestyle [6]. Regular and moderate physical activity is known to contribute to the prevention and management of NCDs [7], the maintenance of mental health [8], and the improvement of overall quality of life [9].

Based on international recommendations, the Romanian Ministry of Health adopted a comprehensive guideline addressing healthy dietary habits (“Guide for healthy eating”), taking into consideration the food types generally available to the Romanian population. The guide summarizes the components of healthy eating in a food pyramid; the base of the pyramid consists of daily physical activity, adequate water consumption, and flour-based foods, exemplified by bread, cereals, rice, or pasta. These basic recommendations are complemented by a plentiful intake of fruits and vegetables, and a moderate intake of dairy products and low-fat meat. Finally, the top of the pyramid suggests the limited consumption of saturated fats and concentrated sweets [10].

As a result of the social and economic changes in the last few decades, the Western diet became widespread among the Romanian population, partially replacing the more traditional diet, based mainly on foods with a high caloric value, such as fatty meats, bread, and dairy products [11]. As neither the traditional, nor the “modern” diet fits the criteria for healthy dietary habits, the Romanian population faces a high prevalence of weight-related conditions. A recent report published by the National Institute of Statistics states that 47.6% of individuals over 18 years of age are overweight, and 10.9% are obese [12], leading to a high incidence of NCDs. Unfortunately, these values are reflected in the numbers published by WHO as well, as in 2022, the percentage of deaths from NCDs had risen to 91% and the probability of premature mortality from NCDs to 21% [1].

Studies focusing on the Romanian population have evaluated health status, eating habits, and attitudes towards a healthy lifestyle. The main findings of these studies reflect that there is a concerning pattern evident in the general population, characterized by the overconsumption of animal products, primarily meat and dairy products, as well as bread and pastry items. At the same time, many diets have limited diversity and are lacking sufficient quantities of fruits and vegetables [11,13,14]. These unhealthy eating habits are exacerbated by a lack of physical activity, meal skipping, and deficient hydration [14,15]. Of even greater concern is the fact that low adherence to a healthy lifestyle is prevalent among the younger age groups [14,16], which highlights the urgency of addressing this issue with effective and targeted health education and healthy lifestyle programs.

Interestingly, studies involving the Romanian population do not report on ethnic minority groups. According to the 2021 national census, approximately 10% of the country’s population belongs to an ethnic group, the two largest minorities consisting of Hungarians (approximately 5%) and Roma (approximately 3%) [17]. While there are numerous overlapping cultural and traditional aspects between these ethnicities, some divergences are to be expected, the attitude towards a healthy lifestyle being one of them. This hypothesis was partially demonstrated by Nedo and Paulik, who found that there are significant differences between the populations of Romania and Hungary regarding their dietary habits. The authors reported that in Hungary obesity is primarily associated with a medium education level, while in Romania, an unhealthy diet seems to be the major determining factor [18].

Our study proposed to evaluate the dietary habits of the ethnic Hungarian minority living in Romania, focusing on dietary patterns, food purchasing choices, and nutritional diversity. By comparing our findings with the information available about the country’s general population, we aimed to identify key differences in nutritional behavior, highlighting potential challenges and necessary interventions for the minority population.

## 2. Materials and Methods

### 2.1. Study Design

A cross-sectional, observational, questionnaire-based study was carried out during July–August 2024, to evaluate the food purchasing and dietary habits of ethnic Hungarian adults living in Romania. Data collection was carried out using the Google Forms platform, through an anonymous online questionnaire. The questionnaire was disseminated on social media and various online groups. Participation in the study was voluntary. The respondents were informed in the introduction of the questionnaire regarding the aim and scope of the study, the anonymous nature of the responses, as well as compliance with the General Data Protection Regulation (GDPR). The study was approved by the Ethics Committee for Scientific Research of the George Emil Palade University of Medicine, Pharmacy, Science, and Technology of Targu Mures (Decision No. 3261/25.06.2024).

### 2.2. Questionnaire Structure

The questionnaire consisted of two main sections. The first section included 5 questions (I–V) regarding anthropometric and socio-demographic data. Collected parameters included age, gender, current or completed studies, height, and weight. Age was divided into three categories: young adults (18 to 30 years), middle-aged adults (30 to 50 years), and older adults (aged over 50). Current or completed studies were also divided into three categories, namely secondary education only, pursuing undergraduate studies, and undergraduate or graduate degree, respectively. Self-reported data on weight and height were used to calculate the body mass index (BMI) by dividing the person’s weight (in kilograms) by the square of their height (in meters).

The second section of the questionnaire consisted of 18 questions addressing six key topics related to dietary patterns (Q1–Q3), purchasing habits (Q4–Q7), labeling information (Q8–Q10), dietary preferences (Q11–Q16), water consumption (Q16), and the use of dietary supplements (Q17–Q18). A complete questionnaire is presented in Appendix A.

### 2.3. Data Analysis

Data collection and evaluation was performed using Microsoft Excel (Microsoft Corporation, Redmond, WA, USA). Statistical analysis was carried out using GraphPad v3.06 (GraphPad Software Inc., Boston, MA, USA), and statistical significance was considered at *p* < 0.05. A 95% confidence interval with a 6% margin of error was used to ensure reliability. Multivariate data analysis (MVDA) was performed using SIMCA^®^ 18 (Sartorius Stedim Biotech, Göttingen, Germany). MVDA consisted of principal component analysis (PCA-X) models to establish pattern recognition and data clustering, as well as orthogonal partial least squares discriminant analysis (OPLS-DA) models to examine class differences. Loading column plots highlighted variables with discriminatory power, enabling comparison between groups, representing the direction and magnitude of loading coefficients.

## 3. Results

### 3.1. Anthropometric and Demographic Data

The questionnaire was completed by 247 individuals. The most represented age category was that of young adults between 18 and 30 years of age, as 56.3% (*n* = 139) of all the respondents belonged to this age group. They were followed by adults between 30 and 50 years of age, accounting for 24.7% (*n* = 61) of all the data, and older adults aged above 50 years, covering 19.0% (*n* = 47) of the respondents. Considering the gender distribution of the respondents, 67.2% were females (*n* = 166) and 32.8% were males (*n* = 81). Regarding the calculated body mass index, 54.7% (*n* = 135) of the respondents had a normal body weight, while 23.5% (*n* = 58) were overweight. Extremities, like undernourished and obese individuals accounted for 8.1% (*n* = 20) and 13.8% (*n* = 34), respectively. The level of education of the respondents showed the following distribution: 16.2% (*n* = 40) of the respondents had completed secondary education, 38.1% (*n* = 94) were pursuing undergraduate studies, and 45.7% (*n* = 113) had an undergraduate or graduate degree.

### 3.2. Dietary Patterns

At the moment of the evaluation, 40 individuals stated that they were following a specific diet (Q1), among which intermittent fasting was the first choice. Additionally, 78 respondents reported that they had followed some type of diet in the past (Q2), with an overlap of 22 respondents who had previously followed and were also following a dietary pattern at the time of the study. Of the 40 respondents who were maintaining a dietary pattern at the time of data collection, only 16 were advised to do so by a dietitian or nutritionist. An χ^2^ test of independence was performed to examine the relationships between age and the responses given to Q1. The relationship between these variables was significant, χ^2^ (2, *N* = 247) = 10.898, *p* = 0.0043. Fisher’s exact test revealed a statistically significant difference between older adults and both young adults under the age of 30 and adults between the ages of 30 and 50. Noticeable differences were also observed between the educational levels of the respondents and the diets followed (χ^2^ (2, *N* = 247) = 10.027, *p* = 0.0066), with respondents with undergraduate or graduate degrees being more likely to follow some type of diet than those pursuing undergraduate studies or those with secondary education only. No statistically significant differences were evident for BMI or gender in terms of dietary patterns followed at the time of the study. There were also no statistically relevant differences between any evaluated parameters in terms of previously adopted diets.

**Figure 1:**
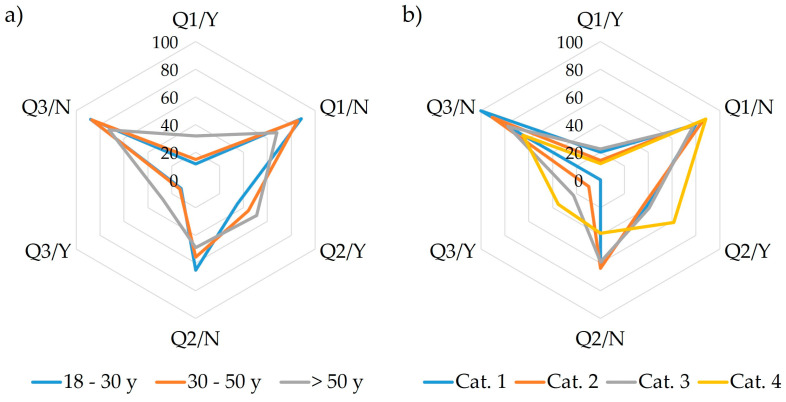
SeDeM plot indicating the frequency of answers given to Q1–Q3 regarding the evaluation of dietary patterns, (**a**) according to age and (**b**) according to BMI categories. Q1—Are you currently following a specific diet? Q2—Have you followed a specific diet in the past? Q3—Have you ever consulted a dietitian or nutritionist for dietary advice or meal planning? Y—yes; N—no.

Question 3 examined if the respondents have consulted a dietitian or nutritionist for dietary advice. The responses revealed differences related to age and educational level, as persons over the age of 30 and generally the graduated, employed respondents had already sought medical care for weight management. It was also observed that with increasing BMI, respondents were more likely to consider a specific diet and rely on professional medical healthcare (Figure 1). The statistical interpretation of the obtained results for Q1–Q3 are presented in Table 1.

**Figure 2:**
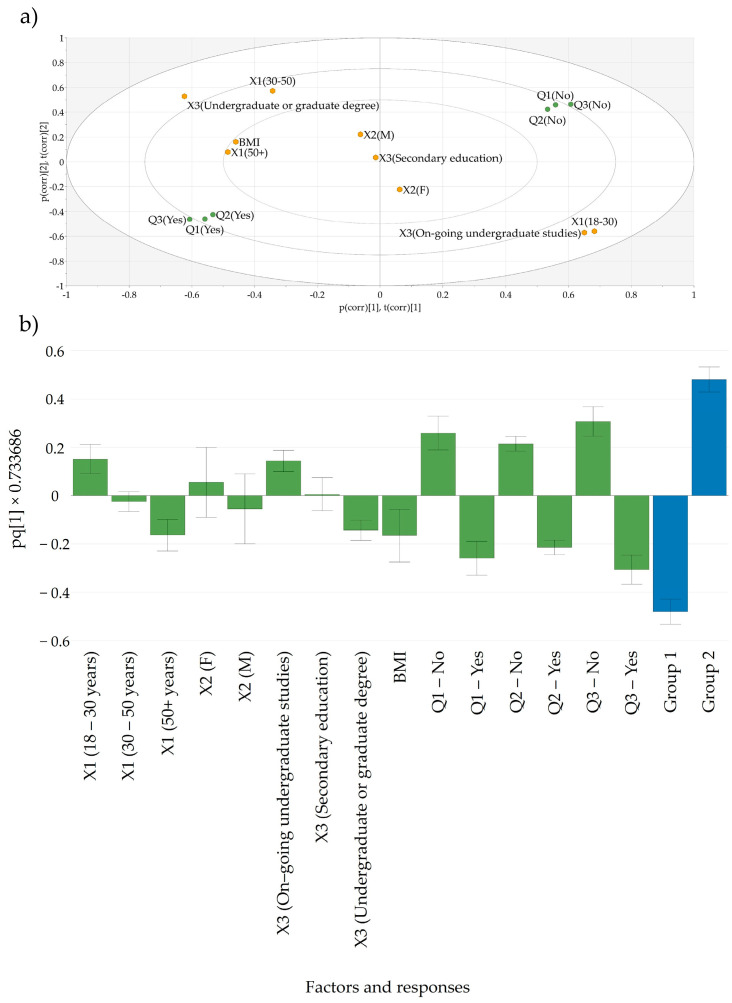
(**a**) Biplot showing the distribution responses given to Q1–Q3 according to the input factors and (**b**) the loading plot of the constructed OPLS–DA model constructed for Q1–Q3 indicating differences based on data clustering. Q1—Are you currently following a specific diet? Q2—Have you followed a specific diet in the past? Q3—Have you ever consulted a dietitian or nutritionist for dietary advice or meal planning? X1—age; X2—gender; X3—level of education; BMI—body mass index.

The PCA–X model built for the evaluation of lifestyle habits (Q1–Q3) in terms of followed dietary patterns revealed more complex relationships between the anthropometric and demographic data and the responses given to the first three questions. The biplot obtained indicated that adults aged above 30 years and with a higher BMI usually followed a restrictive diet and sought the advice of a medical professional to achieve weight management goals. Conversely, young adults pursuing undergraduate studies tended not to follow diets. No gender-based differences were observed. However, intriguingly, if data were evaluated on the second principal component (vertical direction) it was revealed that a cluster of respondents from each category, i.e., adults with a high BMI and young adults, have given the exactly opposite responses (Figure 2a). To further evaluate this discrepancy, an OPLS–DA model was constructed for these data, separating two distinct groups of the respondents. The created OPLS–DA model captured 65.6% of the variability in the data and has underpinned that older adults with a higher BMI are indeed more likely to follow physician-guided diets compared to the younger population (Figure 2b).

### 3.3. Purchasing Habits

No significant differences were detected between groups regarding food shopping preferences (Q4), as farmer’s markets, local grocery stores, supermarkets, and organic grocery stores were equally chosen as sources for acquisitioning food items in all studied groups. Similarly, no significant differences were observed between groups with regard to the dining habits of the respondents (Q7), with all groups expressing similar tendencies to consume home-cooked meals, takeout, or fast food.

Regarding market preferences for purchasing fruits and vegetables (Q5), statistically significant differences were obtained in an age-, gender-, and education-dependent manner. It was observed that adults aged over 30 years were generally more likely to purchase local products, whereas younger individuals preferred nationwide or imported goods. The differences in terms of market preferences according to gender indicated that male individuals were more inclined to purchase imported products, while female individuals searched for domestic food products. From an educational perspective, respondents with a graduate degree were more likely to purchase local products, while students were more open to nationwide and international markets. The same patterns were observed for the answers given to Q6, regarding the preferences when purchasing other groceries, with noticeable differences observable for the age, gender, and education of the respondents. The statistical analysis of the responses is summarized in Table 2.

**Figure 3:**
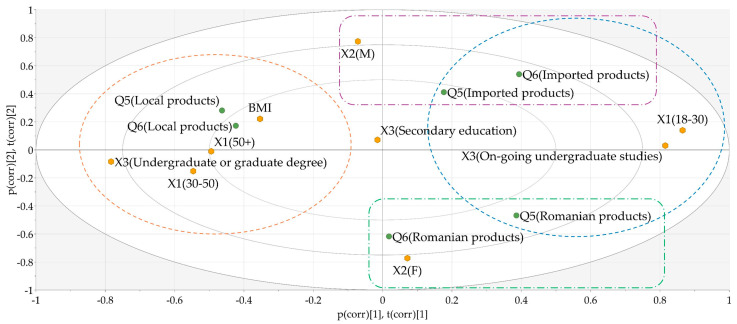
Biplot showing the distribution of responses given to Q5 (When purchasing fruits and vegetables, which do you prefer?) and Q6 (When purchasing other groceries (e.g., dairy, meat, packaged goods), which do you prefer?) according to the input factors. X1—age; X2—gender; X3—level of education; BMI—body mass index. Orange dotted circle—adults 30+ with higher BMI, preferring local products. Blue dotted circle—younger individuals, neutral for fruits/vegetables but preferring imported groceries. Violet dotted rectangle—males, favoring imported products. Green dotted rectangle—females, relying on domestic products.

The obtained biplot of the PCA–X model illustrates that adults aged 30 and above, with higher BMI values generally preferred local products when purchasing fruits, vegetables, or food products (Figure 3, the cluster in the orange dotted circle). On the other hand, younger persons, who would have considered native or imported products alike when purchasing fruits and vegetables, indicated that imported products would have been their first choice for other groceries (Figure 3, the cluster in the blue dotted circle). In terms of gender differences, it was revealed that male respondents usually preferred to purchase imported products (Figure 3, the violet dotted rectangle), while female respondents mostly relied on domestic products (Figure 3, the green dotted rectangle).

### 3.4. Food Composition and Labeling

There were no important associations between the anthropometric and demographic data of the questionnaire participants and the answers given to Q8 and Q10, namely the habit of reading the ingredients list of products before purchase and searching for additional information about additives, respectively. However, for the respondents who did consult it, the information provided on the food label influenced their purchasing habits (Q9), and this was usually observed in an age- and education-dependent manner. The statistical results for these responses are listed in Table 3.

**Figure 4:**
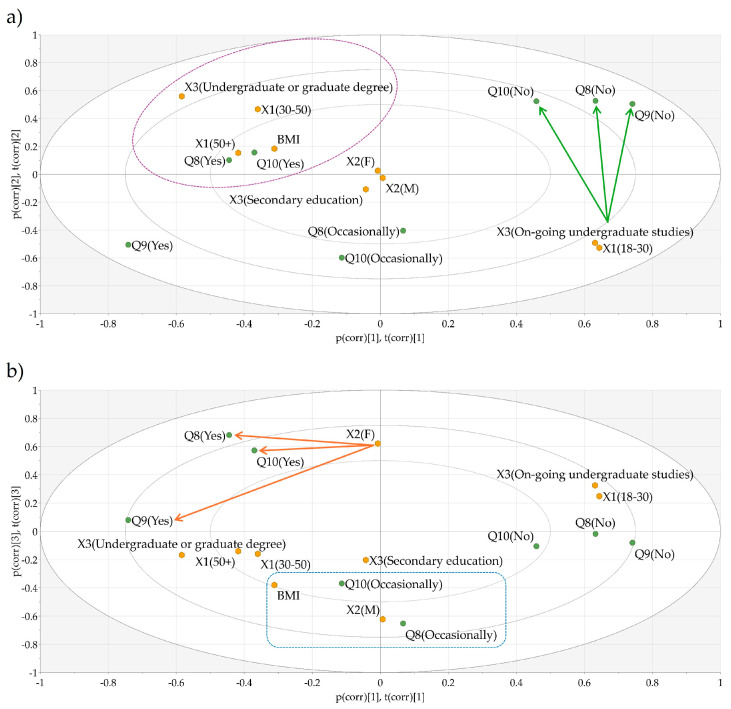
(**a**) Biplot illustrating factor-to-response interactions based on age, weight, and education differences. (**b**) Biplot illustrating gender-related differences regarding the answers given to Q8–Q10. Q8—Do you read the composition or ingredients list of products before purchasing them?; Q9—Does the information about the composition or ingredients of a product influence your decision to purchase it?; Q10—Do you search for information about additives listed in the ingredients of a product before purchasing it?; X1—age; X2—gender; X3—level of education; BMI—body mass index.

Evaluating all the responses given to Q8–Q10, further in-depth relationships were elucidated as illustrated in Figure 4. Similarly to the previous questions, positive answers to Q8 and Q10 were usually given by adults above the age of 30 with a high BMI (Figure 4a, the purple dotted oval), whereas the younger generation generally gave negative answers to these questions (Figure 4a, the green arrows). Regarding Q9, all age groups were influenced by the information on the food labels to a certain extent; however, the older population was more inclined to base their purchasing decision on this information. Further differences were identified in a gender-dependent manner, as shown in Figure 4b. Female respondents were more precautious when purchasing goods, giving mostly positive answers for all three questions (Figure 4b, the orange arrows). On the other hand, male respondents, generally with a higher BMI, were only occasionally focused on reading the composition of foods or searching for supplementary information regarding the listed ingredients, and their choice was usually not influenced by labeling information (Figure 4b, blue dotted rectangle).

### 3.5. Dietary Habits

**Figure 5:**
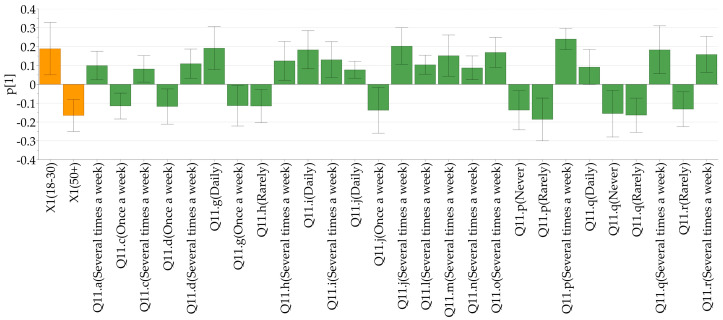
Loading plot illustrating the differences in the nutritional habits of young (18–30 years) and older (>50 years) respondents. Q11—How often do you consume the following types of products? Q11a—Fruits; Q11c—Baked goods; Q11d—Grains; Q11g—Meats; Q11h—Fish and seafood; Q11i—Processed meats; Q11j—Pasta; Q11l—Nuts and seeds; Q11m—Animal fats; Q11n—Vegetable fats; Q11o—Sweets; Q11p—Salty snacks and fast-food; Q11q—Sugary soft drinks; Q11r—Alcohol.

**Figure 6:**
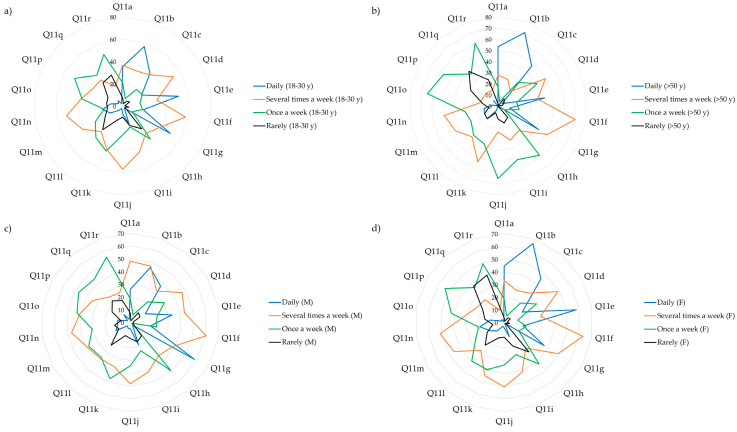
SeDeM plot illustrating the frequency of responses given by the respondents regarding the consumption of different products (Q11). (**a**) 18–30 years, (**b**) >50 years, (**c**) male and (**d**) female respondents. Q11a—Fruits; Q11b—Vegetables; Q11c—Baked goods; Q11d—Grains; Q11e—Dairy products; Q11f—Eggs; Q11g—Meats; Q11h—Fish and seafood; Q11i—Processed meats; Q11j—Pasta; Q11k—Legumes; Q11l—Nuts and seeds; Q11m—Animal fats; Q11n—Vegetable fats; Q11o—Sweets; Q11p—Salty snacks and fast-food; Q11q—Sugary soft drinks; Q11r—Alcohol.

The differences in the frequency of the consumption of various food types (Q11) is illustrated in Figure 5 (only significant relationships are shown). The loading plot of the PCA–X model constructed to evaluate the differences between the responses given has shown some intriguing insights into the nutritional habits of the respondents. The factor-to-response relationships on the PC1 describe the differences in an age-dependent manner. Younger respondents reported daily consumption of meat and meat products (11g and 11i), pasta (11j), and soft drinks (11q), whereas the older population consumed meat and meat products or pasta only once a week, and sugary soft drinks rarely or never. Furthermore, fruits (11a), baked goods (11c), grains (11d), seafood (11h), seeds (11l), fats (11m, 11n), sweets (11o), snacks and fast food (11p), and alcohol (11r) were consumed several times per week by the younger generations, while being less characteristic for respondents older than 50 years. Figure 6 depicts the dietary preferences by age and gender categories.

**Figure 7:**
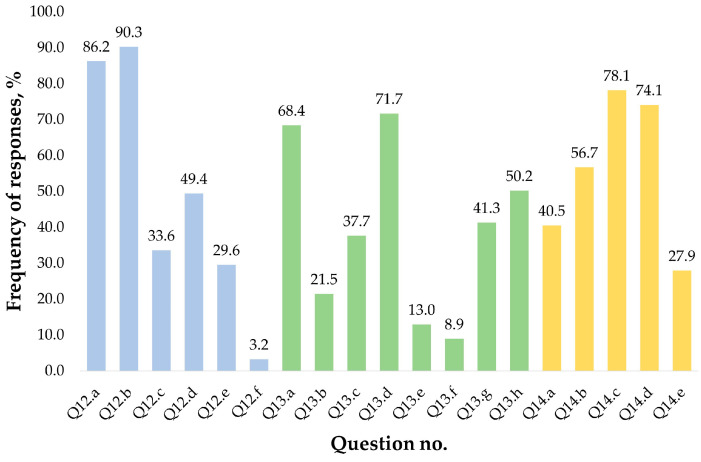
Frequency of responses to questions regarding food sources for macronutrients. Q12 (blue bars)—Which of the following protein sources do you use most frequently in your diet? (Q12a—Meat and meat products; Q12b—Dairy products; Q12c—Seafood; Q12d—Legumes; Q12e—Seeds; Q12f—Soybeans); Q13 (green bars)—Which of the following carbohydrate sources do you use most frequently in your diet? (Q13a—Baked goods; Q13b—Rice; Q13c—Pasta; Q13d—Potato; Q13e—Sweets and soda; Q13f—Legumes; Q13g—Fruits; Q13h—Vegetables); Q14 (yellow bars)—Which of the following lipid sources do you use most frequently in your diet? (Q14a—Fruits and vegetables; Q14b—Seeds; Q14c—Vegetable oils; Q14d—Animal fats; Q14d—Oily fishes).

Concerning the major food sources for macronutrients (Q12—proteins, Q13—carbohydrates, and Q14—lipids) no statistically significant differences were determined between groups. Regarding the most important protein sources, dairy, meat, and meat products were consumed by most of the respondents, although almost half of them also marked legumes as an important source of proteins in their diet. Seafood and various seeds were specified by one-third of all study participants, while soybeans were used by only eight persons. The most common source of carbohydrates was potatoes and baked goods, marked by two-third of the respondents. These were followed by fruits and vegetables with nearly half of the responses marking these products, and almost one-third of the respondents noted pasta as a source of carbohydrates in their diet. Rice, legumes, sweets, and soda were the least chosen as sources of carbohydrates. The most frequently selected lipid sources were vegetable oils and animal fats; however, various oily seeds were also considered by more than half of the respondents. Fruits and vegetables (such as soybeans) or fish were the least marked lipid intake sources. The frequency of responses to questions Q12–Q14 is presented in Figure 7.

**Figure 8:**
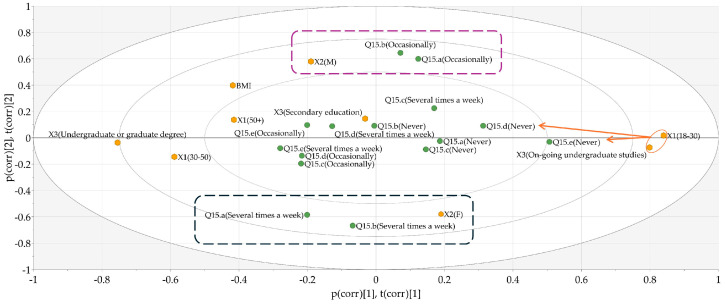
Biplot showing the distribution of responses according to anthropometric and demographic data of the respondents. Q15—How often do you use the following types of fats for cooking? (Q15a—Butter; Q15b—Sunflower oil; Q15c—Olive oil; Q15d—Other vegetable oils; Q15e—Lards); X1—age; X2—gender; X3—level of education; BMI—body mass index. Orange arrows—younger individuals were less likely to use oils and animal lards. Blue & violet dotted rectangles—females preferred butter and sunflower oil.

Regarding Q15, referring to the use of different types of fats for cooking, relevant differences were obtained only for vegetable oils other than sunflower and olive oil (Q15d), and animal lards (Q15e). As depicted in Figure 8, age-, gender- and education-level differences were identified between the groups. The statistical results are presented in Table S1. The younger population was less willing to use different types of oils and animal lards (Figure 8, orange arrows), compared to adults above 30 years old. Although classical statistical analysis revealed that the BMI of respondents was not directly correlated with the type of fats used for cooking, PCA indicated that older individuals with a higher BMI were more likely to use animal lards as cooking fats. Taking into consideration the gender differences, beside the statistically significant differences regarding using animal lards, it was also noted that female respondents were more likely to use butter and sunflower oil compared to male respondents (Figure 8, blue and violet dotted rectangles); however, these divergences might be attributed to the frequency difference in preparing meals between genders.

### 3.6. Water Intake

**Figure 9:**
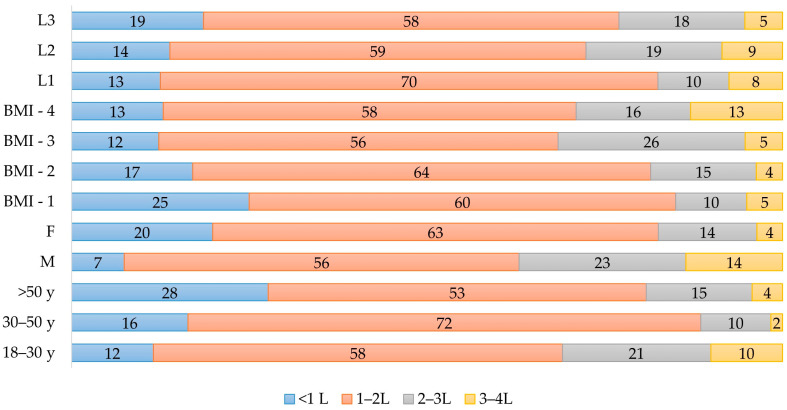
The daily water consumption of the respondents. F—female; M—male; L1—secondary education; L2—pursuing undergraduate studies; L3—undergraduate or graduate degree; BMI—body mass index.

The amount of water consumed daily (Q16) differed significantly between the age groups (Table 4), where at the extremities of the respondents were individuals over 50 years with almost one-third of them consuming less than 1 L of water daily. By contrast, almost the same percentage of young respondents declared that they consume more than 3 L of water daily. Noticeable differences were observed in a gender-dependent manner as well, as female respondents tended to consume less water than their male counterparts. Also, significant differences were obtained for the BMI of the surveyed individuals. Overweight and obese persons tended to consume more water than normal-weight or underweight individuals. No statistically relevant differences were observed based on the level of education. The frequency of water consumption among the respondents is depicted in Figure 9.

### 3.7. Use of Dietary Supplements

The use of various dietary supplements was marked by 57.5% (*n* = 142) of the respondents, of which 132 have noted vitamins and minerals, 71 proteins, 60 immunostimulants, and 20 creatin for this category. The statistical results are presented in Table 5. Differences regarding the use of dietary supplements were observed in terms of BMI, when comparing the normal-weight category with the overweight and obese categories. As such, more than two-thirds of the normal-weight individuals consumed dietary supplements, compared to less than 50% of the overweight and obese respondents.

### 3.8. Global Analysis of the Responses

**Figure 10:**
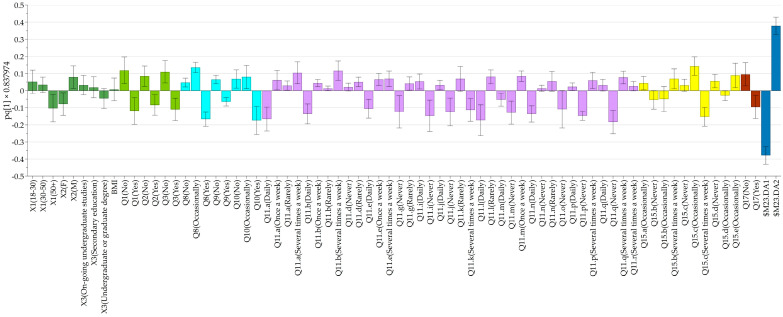
Loading plot of the general OPLS-DA model accounting for all gathered information.

Finally, an overall analysis of the responses was conducted by hierarchical clustering analysis (HCA), revealing data clustering at several levels. The obtained loading plot of the created OPLS-DA model is presented in Figure 10. Two noticeable clusters were identified comprising elderly, female (DA1) and younger, male respondents (DA2). No discrimination was observed in terms of BMI and educational level. The correlations obtained underpinned the observations made in the previous sections, i.e., the interest of the elderly respondents in a healthier diet, driven by conscious food purchasing habits, and rational food consumption by omitting high-fat, high-calorie, and unhealthy snacks compared to the younger generation.

## 4. Discussion

### 4.1. Dietary Patterns

Specific dietary patterns or restrictive diets are frequently adopted by the general population either as a healthy lifestyle choice, or a method for weight loss. Indeed, appropriate dietary patterns have been linked with weight loss [19], improved glycaemic control [20], and a decreased risk of cardiovascular diseases [21] or cancer [22,23]. There are numerous well-known dietary patterns, such as vegetarian, vegan, ketogenic, paleolithic, intermittent fasting, and carnivore diet, among others. However, only a few of them adhere to the current recommended dietary guidelines and have scientifically proven health benefits. The main dietary patterns with evidence-based benefits are the Dietary Approach to Stop Hypertension (the DASH diet), the Mediterranean diet, and vegetarianism, if the latter includes sufficiently varied food groups [24]. The adoption of a dietary pattern for health-related reasons should be made at the recommendation of a nutrition expert [25]; however, due to cultural and socio-economic reasons, the general population in Romania is less likely to seek advice from a dietitian [26].

Our results show that almost one-third of the respondents (31.6%) have at one point adopted a specific dietary pattern, but only 16% were adhering to one at the time of the study. Of those maintaining a restrictive diet, less than half have consulted a nutrition specialist. The dietary patterns followed by the respondents, in order of frequency, were intermittent fasting, vegetarian and vegan diets, hypocaloric and sugar-free diets, ketogenic, paleolithic, and Rina’s 90-day diet. Intriguingly, the Mediterranean diet was only mentioned twice, while the DASH diet was not adopted by any of the respondents. Furthermore, only six participants were following a personalized dietary plan. These results indicate that while specific dietary patterns are relatively common among the Hungarian ethnic group, the decision to follow them is not anchored in factual knowledge regarding their health benefits.

Regarding the correlation between responses and the studied factors, a trend could be observed wherein adults over 30 years of age with higher BMI values were more likely to have adopted some form of restrictive diet and had sought advice from a healthcare professional for weight management. Although a study conducted on the general population of the country found that restrictive diets are more frequently adopted by overweight and obese individuals, most commonly women [14], our study did not find a direct correlation between gender and dietary patterns.

### 4.2. Purchasing Habits

The decision regarding where to source food products for personal consumption is influenced by several factors such as perceived health benefit, financial considerations, organoleptic aspects, brand perception, and sustainability [27]. Locally and domestically produced fruits, vegetables, and other food items are generally considered to be healthier and more sustainable than their imported counterparts, partially owning to reduced carbon footprint and a reduced use of additives in the form of preservatives. On the other hand, these products are often considered to be more expensive, especially if they are marketed as organic products [28]. Studies show that Romanian consumers are increasingly conscious of the environmental impact of their purchasing choices and tend to adopt sustainable purchasing and consumption behaviors [29]. The main factors that influence purchasing behavior have been found to be the quality and taste of the products, as well as their health benefits. Among the factors limiting the adoption of a more sustainable purchasing behavior were financial considerations, the low availability of products, and misleading or unclear labeling [30].

Based on the responses, we concluded that the studied population can be clustered depending on their purchasing habits. Young adults (age 18–30), especially those pursuing graduate degrees, were inclined to purchase imported food products, except for fruits and vegetables. However, individuals over the age of 30 with a higher level of education were prone to purchase locally produced fruits, vegetables, and other food products. Gender differences in purchasing habits were also found, with female respondents more likely to buy domestic products, while their male counterparts were more interested in imported products.

Voinea et al. explored the behavior of the Romanian population regarding food choices and found that the respondents could be grouped into two main clusters. The first cluster consisted of consumers with a lower BMI, showing concern for healthy eating and the use of natural ingredients and organic farming. The second cluster included consumers mostly interested in the organoleptic features of food products, taking little notice of their nutritional value. As expected, those in the second cluster expressed significantly higher BMI values, most likely due to their unbalanced diets [31]. A questionnaire-based study conducted in Hungary found that only approximately a quarter of the respondents were conscious consumers that make their purchasing decisions based on the nutritional value of food products and prefer domestic products [32]. In another study, the same author group found that the main consumers of domestic products in Hungary were the older adults (“Generation X”), while young adults (“Generation Z”) showed a neutral attitude towards these products, attributed to the lack of a traditional value system [33].

This set of questions also evaluated the source of prepared meals consumed on a daily basis. Although no statistically significant correlation could be found between dining habits and the studied parameters, there was clear indication that the ethnic Hungarian population in Romania vastly preferred home-cooked meals, with 96% of the respondents marking this category as a source of everyday food intake. On the other hand, approximately 25% of those participating in the survey regularly ate fast food-type meals, and only 17% would have considered takeout or restaurant dining. Nastasescu et al. reported a similarly high proportion—approximately 80%—of the Romanian population preferring home-cooked meals [34]. However, these findings might have been influenced by limitations caused by the COVID-19 pandemic during which the study was conducted.

### 4.3. Food Composition and Labeling

The interest of consumers in healthy and sustainable products materialized, among other aspects, in the expectation of “clean labels”, a general term to reflect that a food product does not contain any harmful additives or chemicals [35,36]. A literature review found that the perception of food without artificial additives and natural food was correlated with both demographic data, e.g., age, gender, education, and intrinsic or extrinsic product characteristics, e.g., sensory attributes, type of additives, cost, health concerns, and labeling [36]. At the same time, consumer trust regarding the quality and safety of food products could be gained by providing adequate information on the packaging labels. The information should refer primarily to the intrinsic characteristics of the product, such as composition, production methods, nutritional value, and environmental considerations. Furthermore, specific claims, e.g., organic, environmentally friendly, or those regarding animal welfare, should be appropriately certified. The country or region of origin, along with traceability information, is also an important factor for consumers when evaluating the quality of a product [37,38,39,40].

Based on the responses expressed for the questions related to food labeling, we observed that individuals above the age of 30 and with higher BMI values were more interested in the composition of food products than their younger counterparts. Gender-specific differences were also identified, with female respondents paying more attention to the labeling information and being more influenced by it when purchasing products. A study conducted in Romania highlighted that the segment of the population least concerned with food labeling included young adults with a low education level and insufficient income [41]. Adequate nutritional education is necessary for both adults and children, to raise awareness of the importance of labeling information, food safety, and risk factors [42]. Based on our findings, educational programs targeting Hungarian young adults in Romania are especially important, as this subgroup expressed little to no interest in healthy eating habits.

### 4.4. Dietary Habits

One of the major components of a healthy lifestyle consists of a balanced diet. To reduce the prevalence of diet-related non-communicable diseases, current recommendations include the adequate consumption of fruits and vegetables, whole-grain products, fish rich in polyunsaturated fatty acids (PUFAs), olive oil and other vegetable oils rich in PUFAs, as well as nuts and legumes. The moderate consumption of eggs and dairy products has also been shown to have a positive effect on health. Conversely, large quantities of processed foods, products with a high sugar content, fatty meat, and refined grains should be avoided [6,43].

Several large-scale questionnaire-based studies have been conducted on the general Romanian population, and depicted a concerning situation. In one regard, some of these studies reported that more than half of the respondents had an above-normal body weight, with approximately 30% being overweight and 20% being obese individuals. High BMI values were correlated with unhealthy eating patterns, inadequate diets, and lack of physical activity [15,44]. Gender and education level were also shown to influence attitudes towards healthy eating habits, as female respondents and those with a higher education expressed more concern for a balanced diet [13,16]. The former might be explained by the fact that in most traditional Romanian households, women are responsible for acquiring and preparing meals, while the latter might be attributed to better access to information.

An even more worrisome finding was that the country’s younger population expressed little to no interest in a healthy lifestyle and were prone to the overconsumption of calorie-rich food products, while neglecting the adequate intake of fruits and vegetables [14]. Especially for young adults pursuing undergraduate studies, this lack of interest in a healthy lifestyle might be related to financial considerations, limited free time, as well as peer pressure, e.g., fast food consumption as a social activity.

Regarding consumer behavior towards food products, a study by Balan et al. found that the Romanian population tended to overconsume bread and pastry products, starchy vegetables (primarily potatoes), and meat and meat products. On the other hand, the reported consumption of fruits and vegetables, nuts and seeds, as well as fish and seafood, was below the recommended amount [11]. Intriguingly, another study conducted in the same period reported that fruit and vegetable consumption increased to at least one serving a day, attributing it to a more health-oriented mentality due to the COVID-19 pandemic [34].

Our findings suggest that the ethnic Hungarian population shows similar patterns in food choices. Meat and dairy products represented the major protein sources, being a regular food choice especially among younger adults, while vegetable oils and animal fats accounted for most of the lipid intake. Carbohydrates were most frequently acquired through baked goods and potatoes, followed by fruits and vegetables, but pasta was also selected by almost one-third of the respondents. It is worth mentioning that younger adults marked significantly more food items as part of their frequently consumed food types than individuals over 50. Although these products included both healthy and unhealthy choices, we can conclude that the younger generation has a more diversified diet than the older population.

### 4.5. Water Intake

Adequate hydration is an important part of a healthy lifestyle, as water is an important component of the human body, being involved in blood circulation, metabolic processes, thermoregulation and cellular homeostasis, among other processes [45,46]. According to the Scientific Opinion of the European Food Safety Authority (EFSA) Panel on Dietetic Products, Nutrition, and Allergies (NDA), the recommended daily total water intake (TWI) for adults is 2.5 L for men, and 2.0 L for women. Out of the TWI, 80% should come from fluid intake (water or beverages), namely 2.0 L for men and 1.6 L for women. The guide gives recommendations for both genders in numerous age categories [47]. Studies have found these values to be correct; however, the water homeostasis is influenced by several intrinsic and extrinsic factors, and TWI may vary according to physical activity, health status, climate, diet, and habits [46,48].

Our study revealed that only approximately one-quarter of the respondents reported drinking more than 2 L of water daily. This observation contrasts with results published by Mititelu et al. who found that in their target group, including the general Romanian population, almost half of the participants consumed at least 2 L of water daily [14]. Furthermore, the aforementioned study did not find a correlation between water intake and BMI, while our study showed that overweight and obese individuals tended to consume more water than their normal-weight counterparts. We also found significant differences regarding TWI in a gender- and age-dependent manner, as men and young adults under 30 years showed a higher daily water intake. A worrisome finding was the decreased water consumption in the older age cohort, as roughly one-third of this group reported a daily TWI under 1 L. As the daily TWI for older adults is identical to those of the middle-aged adults, low water consumption puts the older generation at risk of low-intake dehydration [49,50].

### 4.6. Use of Dietary Supplements

Dietary supplements are commonly defined as food products that contain nutrients, such as vitamins, minerals, amino acids, fatty acids, or herbal extracts, among others. They are marketed as an important part of a healthy diet, to complement essential substances ingested through food; however, there is no conclusive evidence of their benefits other than cases of malnutrition or other chronic diseases that demand supplementation of essential nutrients [51,52]. Furthermore, concerns have been raised regarding their quality and subsequent health effects, as dietary supplements are minimally regulated by authorities. Considering that producers are not required to demonstrate the efficacy and safety of these products, there is a high risk of ineffective or contaminated supplements reaching the general population [51,53]. Nevertheless, the consumption of dietary supplements is on the rise globally, with more than 50% of the population of US and at least 30% of Europeans claiming frequent use of these products [54,55]. Aysin et al. proposed even higher numbers, due to the COVID-19 pandemic, with the use of supplements reaching 75.7% in the US and 68.7% in Europe [56]. Age- and gender-related differences have been reported regarding the use of dietary supplements, with older adults and women being more inclined to use these items [57,58].

In our sample population, 57.5% of the respondents declared using or having used dietary supplements. However, there was no observable age- or gender-dependent correlation regarding the use of these products. Interestingly, differences in the use of dietary supplements were observed depending on the BMI of participants, with normal-weight individuals being more likely to use supplements compared to their overweight or obese counterparts. This might be explained with the somewhat incorrect perception that dietary supplements should be part of a healthy diet. Another possible explanation could derive from the study by Fagaras et al., who found that active and very active young Romanian adults have an increased intake of dietary supplements in the form of vitamins and proteins [59].

### 4.7. Promoting Healthy Eating in Young Adults

Several underlying factors might contribute to the limited interest in healthy eating habits among young adults. From a cultural point of view, both Romanian and Hungarian traditional cuisine is characterized by excessive meat consumption, with meat or meat-based products often consumed at all three main meals of the day. Traditional eating habits persist even among young adults, as the general population remains conservative and connected to its cultural heritage. Given the high intake of saturated fats and processed meats, traditional dishes frequently do not correspond with current nutritional recommendations [60,61]. On the other hand, the transition to contemporary dietary habits often consists of adopting the fast food culture. As this is characterized by excessive consumption of high-energy foods, processed products, and sugary beverages, it does not offer a healthy alternative for those adopting it [62,63]. Educational campaigns and health programs should focus on promoting healthy dietary alternatives, considering the characteristics of traditional cuisine, while also highlighting its potential negative health effects.

A study by Muresan et al. showed that the food choices of the general Romanian population are heavily influenced by their financial status and food prices [64]. This trend is particularly evident in young adults pursuing an undergraduate degree, as they often lack a direct source of income [65]. Given that food prices are a primary concern for the Romanian consumers, policies subsidizing healthy foods while taxing unhealthy products could be highly effective.

Health literacy is another aspect that should be considered for this group. Studies conducted on the general population have found a positive association between health literacy and education, while age was negatively associated with health literacy [66,67]. Undoubtedly, integrating nutrition education into school programs, alongside healthy meal programs for children, could encourage the development of lifelong healthy eating habits [67]. Additionally, targeted awareness campaigns should consider the characteristics of specific population groups, such as age-dependent information acquisition methods (e.g., online, newspapers, television advertisements) and potential language barriers in the case of ethnic minorities. These campaigns should involve general practitioners, dietitians and other healthcare professionals to improve the dissemination of information [31,66].

While not specific to young adults, misleading health claims on food labels can also negatively influence purchasing decisions. Regulating food advertising, and enforcing strict labeling requirements for food products could further support informed purchasing decisions [68,69].

Teenagers and young adults are increasingly targeted by various marketing strategies, among which some can be linked with unhealthy dietary choices. Aggressive advertising and targeted online ads, coupled with promotional discounts, can have a notable effect on consumer perception and preferences, influencing the food purchasing decisions, especially for those with financial constraints [68]. Furthermore, with the prevalence of social media, influencers and content creators significantly contribute to the perception of unhealthy food products, often promoting sugary beverages and fast food-type items [70,71]. Considering current trends, promoting credible influencers and lifestyle advisors who advocate a healthy lifestyle could also enhance public engagement, especially in younger adults and teenagers, who are profoundly affected by social media [65,72].

### 4.8. Strengths and Limitations of the Study

To the best of our knowledge, this is the first report on the eating behavior of the ethnic Hungarian population living in Romania, offering a novel insight into the food purchasing and dietary habits of the target group. Using the PCA-X and OPLS-DA models, we were able to identify underlying correlations between variables that were not evident through classical statistical methods. The major limitation of the study was the underrepresentation of some demographic groups, e.g., older adults and those with lower education levels. These groups are known to be less inclined to participate in online questionnaire-based studies. Self-reported questionnaires may be subject to social desirability bias, where respondents modify their responses to align with societal expectations [73]. This can affect the accuracy of various measures, such as BMI calculations based on self-reported weight and height data. Finally, the studies targeting the general Romanian population might have included ethnic Hungarians living in Romania; thus, overlap between target groups could exist.

## 5. Conclusions

In summary, our findings indicate that dietary habits vary significantly in an age-, gender- and education-dependent manner. Specifically, young adults tended to express limited interest in healthy eating habits, characterized by overconsumption of high-calorie foods and neglect of fruits and vegetables, while older adults were more likely to follow dietary patterns and evaluate the composition of food products. On a gender-dependent level, female respondents were notably more likely to adopt healthy eating habits than men. Differences based on education were most evident among respondents with undergraduate or graduate degrees, who expressed greater interest in healthy eating habits when compared to other groups.

Further studies exploring the underlaying correlation between dietary habits and cultural (e.g., traditional cuisine, religious beliefs, food-related beliefs) as well as socio-economic factors (e.g., income level, employment status, food policies) could provide new insights into attitudes towards healthy eating among ethnic Hungarians. Additionally, investigating ethnic identity in a broader social context, such as globalization, urbanization, or migration, could offer novel perspectives on opportunities to promote a healthier lifestyle.

## Figures and Tables

**Table 1:** Statistical data for Q1–Q3 regarding adoption of a diet by the respondents.

Parameter *	Group	Q1 **		Q2		Q3	
		Yes	No	Yes	No	Yes	No
Age	18–30 (1)	16 (6.5%)	123 (49.8%)	33 (17.4%)	62 (32.6%)	17 (6.9%)	122 (49.4%)
	30–50 (2)	9 (3.6%)	52 (21.0%)	23 (12.1%)	29 (15.3%)	8 (3.2%)	53 (21.4%)
	50+ (3)	15 (6.1%)	32 (13.0%)	22 (11.6%)	21 (11.0%)	13 (5.3%)	34 (13.8%)
Statistical evaluation ***		*χ*^2^ (2, *N* = 247) = 10.898, ***p* = 0.004**, for all age categories *p* = 0.642, group 1 vs. group 2 ***p* = 0.003**, group 1 vs. group 3 ***p* = 0.039**, group 2 vs. group 3		*χ*^2^ (2, *N* = 190) = 3.599, *p* = 0.1654, for all age categories *p* = 0.289, group 1 vs. group 2 ***p* = 0.090**, group 1 vs. group 3 *p* = 0.540, group 2 vs. group 3		*χ*^2^ (2, *N* = 247) = 6.744, ***p* = 0.034**, for all age categories *p* = 0.821, group 1 vs. group 2 ***p* = 0.020**, group 1 vs. group 3 *p* = 0.085, group 2 vs. group 3	
BMI	Cat. 1	4 (1.6%)	16 (6.5%)	5 (2.6%)	8 (4.2%)	0 (0%)	20 (8.1%)
	Cat. 2	19 (7.7%)	116 (47.0%)	37 (19.5%)	65 (34.2%)	13 (5.3%)	122 (49.4%)
	Cat. 3	13 (5.3%)	45 (18.2%)	20 (10.5%)	29 (15.3%)	13 (5.3%)	45 (18.2%)
	Cat. 4	4 (1.6%)	30 (12.1%)	16 (8.4%)	10 (5.3%)	12 (4.8%)	22 (8.9%)
Statistical evaluation ***		*χ*^2^ (3, *N* = 247) = 2.805, *p* = 0.423 Normal weight vs. overweight and obese, *p* = 0.456		*χ*^2^ (3, *N* = 190) = 5.508, *p* = 0.138 Normal weight vs. overweight and obese, *p* = 0.125		*χ*^2^ (3, *N* = 247) = 19.625, ***p* < 0.001** Normal weight vs. overweight and obese, ***p* < 0.001**	
Gender	F	28 (11.3%)	138 (55.9%)	58 (30.5%)	20 (10.5%)	24 (9.7%)	142 (57.5%)
	M	12 (4.9%)	69 (27.9%)	70 (36.9%)	42 (22.1%)	14 (5.7%)	67 (27.1%)
Statistical evaluation		*p* = 0.718		*p* = 0.115		*p* = 0.577	
Educational level	Level 1	2 (0.8%)	38 (15.4%)	15 (7.9%)	17 (8.9%)	7 (2.8%)	33 (13.4%)
	Level 2	11 (4.5%)	83 (33.6%)	22 (11.6%)	39 (20.5%)	9 (3.6%)	85 (34.4%)
	Level 3	27 (10.9%)	86 (34.8%)	41 (21.6%)	56 (29.5%)	22 (8.9%)	91 (36.9%)
Statistical evaluation		*χ*^2^ (2, *N* = 247) = 10.027, ***p* = 0.0066** *p* = 0.343, level 1 vs. level 2 ***p* = 0.009**, level 1 vs. level 3 ***p* = 0.030**, level 2 vs. level 3		*χ*^2^ (2, *N* = 190) = 1.134, *p* = 0.567 *p* = 0.375, level 1 vs. level 2 *p* = 0.685, level 1 vs. level 3 *p* = 0.507, level 2 vs. level 3		*χ*^2^ (2, *N* = 247) = 4.023, ***p* < 0.001** ***p* < 0.001**, level 1 vs. level 2 *p* = 1.000, level 1 vs. level 3 *p* = 0.052, level 2 vs. level 3	

* Age: (1)—18–30 years, (2)—30–50 years, (3)—above 50 years; BMI: Cat. 1 < 18.5, BMI Cat. 2 = 18.5–24.9, BMI Cat. 3 = 25.0–29.9, BMI Cat. 4 > 30; Gender: F—female, M—male; Educational level: Level 1—secondary education, Level 2—ongoing undergraduate studies, Level 3—undergraduate or graduate degree. ** Q1—Are you currently following a specific diet? Q2—Have you followed a specific diet in the past? Q3—Have you ever consulted a dietitian or nutritionist for dietary advice or meal planning? *** *p*–values indicate the results of Fischer’s exact test. Values indicated in bold denote statistically significant differences.

**Table 2:** Statistical data for Q5–Q6 regarding the purchasing habits of the respondents.

Parameter	Group	Q5			Q6		
		Local	Romanian	Imported	Local	Romanian	Imported
Age	18–30 (1)	56 (22.7%)	74 (30.0%)	9 (3.6%)	22 (8.9%)	68 (27.6%)	49 (19.8%)
	30–50 (2)	36 (14.6%)	23 (9.3%)	2 (0.8%)	17 (6.9%)	34 (13.8%)	10 (4.0%)
	50+ (3)	31 (12.5%)	14 (5.7%)	2 (0.8%)	20 (8.1%)	23 (9.3%)	4 (1.6%)
Statistical evaluation		*χ*^2^ (4, *N* = 247) = 12.206, ***p* = 0.016**, for all categories *χ*^2^ (2, *N* = 200) = 6.129, ***p* = 0.047**, group 1 vs. group 2 *χ*^2^ (2, *N* = 186) = 9.323, ***p* = 0.010**, group 1 vs. group 3 *χ*^2^ (2, *N* = 108) = 0.760, *p* = 0.684, group 2 vs. group 3			*χ*^2^ (4, *N* = 247) = 23.947, ***p* < 0.0001**, for all categories *χ*^2^ (2, *N* = 200) = 8.650, ***p* = 0.013**, group 1 vs. group 2 *χ*^2^ (2, *N* = 186) = 19.925, ***p* < 0.0001**, group 1 vs. group 3 *χ*^2^ (2, *N* = 108) = 3.176, *p* = 0.204, group 2 vs. group 3		
BMI	Cat. 1	8 (3.2%)	11 (4.4%)	1 (0.4%)	2 (0.8%)	12 (4.8%)	6 (2.4%)
	Cat. 2	71 (28.8%)	59 (23.9%)	5 (2.0%)	32 (12.9%)	64 (25.9%)	39 (15.8%)
	Cat. 3	31 (12.6%)	23 (9.3%)	4 (1.6%)	13 (5.3%)	32 (13.0%)	13 (5.3%)
	Cat. 4	13 (5.3%)	18 (7.3%)	3 (1.2%)	12 (4.9%)	17 (6.9%)	5 (2.0%)
Statistical evaluation		*χ*^2^ (6, *N* = 247) = 4.746, *p* = 0.577 *χ*^2^ (2, *N* = 227) = 1.833, *p* = 0.400, normal weight vs. OW + OB			*χ*^2^ (6, *N* = 247) = 6.921, *p* = 0.328 *χ*^2^ (2, *N* = 227) = 2.533, *p* = 0.282, normal weight vs. OW + OB		
Gender	F	80 (32.4%)	81 (32.8%)	5 (2.0%)	40 (16.2%)	92 (37.2%)	34 (13.8%)
	M	43 (17.4%)	30 (12.2%)	8 (3.2%)	19 (7.7%)	33 (13.4%)	29 (11.7%)
Statistical evaluation		*χ*^2^ (2, *N* = 247) = 6.801, ***p* = 0.033**			*χ*^2^ (2, *N* = 247) = 7.337, ***p* = 0.026**		
Educational level	Level 1	18 (7.3%)	20 (8.1%)	2 (0.8%)	11 (4.4%)	18 (7.3%)	11 (4.4%)
	Level 2	37 (15.0%)	49 (19.9%)	8 (3.2%)	12 (4.9%)	46 (18.6%)	36 (14.6%)
	Level 3	68 (27.5%)	42 (17.0%)	3 (1.2%)	36 (14.6%)	61 (24.7%)	16 (6.5%)
Statistical evaluation		*χ*^2^ (4, *N* = 247) = 10.862, ***p* = 0.028**, for all study levels *χ*^2^ (2, *N* = 134) = 0.705, *p* = 0.703, level 1 vs. level 2 *χ*^2^ (2, *N* = 153) = 2.908, *p* = 0.234, level 1 vs. level 3 *χ*^2^ (2, *N* = 207) = 10.306, ***p* = 0.006**, level 2 vs. level 3			*χ*^2^ (4, *N* = 247) = 20.443, ***p* < 0.001**, for all study levels *χ*^2^ (2, *N* = 134) = 2.134, *p* = 0.102, level 1 vs. level 2 *χ*^2^ (2, *N* = 153) = 3.624, *p* = 0.163, level 1 vs. level 3 *χ*^2^ (2, *N* = 207) = 20.222, ***p* < 0.0001**, level 2 vs. level 3		

Values indicated in bold denote statistically significant differences. Age: (1)—18–30 years, (2)—30–50 years, (3)—above 50 years; BMI: Cat. 1 < 18.5, BMI Cat. 2 = 18.5–24.9, BMI Cat. 3 = 25.0–29.9, BMI Cat. 4 > 30, OB—obese, OW—overweight; Gender: F—female, M—male; Educational level: Level 1—secondary education, Level 2—ongoing undergraduate studies, Level 3—undergraduate or graduate degree. Q5—When purchasing fruits and vegetables, which do you prefer? Q6—When purchasing other groceries (e.g., dairy, meat, packaged goods), which do you prefer?

**Table 3:** Statistical data of Q8–Q10 regarding the food composition and labeling.

Parameter	Group	Q8			Q9		Q10		
		Yes	Occ.	No	Yes	No	Yes	Occ.	No
Age	18–30 (1)	51 (20.6%)	71 (28.8%)	17 (6.9%)	111 (45.0%)	28 (11.3%)	24 (9.7%)	72 (29.2%)	43 (17.4%)
	30–50 (2)	26 (10.5%)	31 (12.6%)	4 (1.6%)	52 (21.1%)	9 (3.6%)	14 (5.7%)	28 (11.3%)	19 (7.7%)
	50+ (3)	20 (8.1%)	25 (10.1%)	2 (0.8%)	45 (18.2%)	2 (0.8%)	14 (5.7%)	24 (9.7%)	9 (3.6%)
Statistical evaluation		*χ*^2^ (4, *N* = 247) = 3.634, *p* = 0.458, for all age categories *χ*^2^ (2, *N* = 200) = 1.687, *p* = 0.430, gr. 1 vs. gr. 2 *χ*^2^ (2, *N* = 186) = 2.533, *p* = 0.282, gr. 1 vs. gr. 3 *χ*^2^ (2, *N* = 108) = 0.282, *p* = 0.869, gr. 2 vs. gr. 3			*χ*^2^ (2, *N* = 247) = 6.734, *p* = 0.035, for all gr. *p* = 0.432, gr. 1 vs. gr. 2 ***p* = 0.010**, gr. 1 vs. gr. 3 *p* = 0.109, gr. 2 vs. gr. 3		*χ*^2^ (4, *N* = 247) = 4.917, *p* = 0.296, for all age categories *χ*^2^ (2, *N* = 200) = 1.017, *p* = 0.602, gr. 1 vs. gr. 2 *χ*^2^ (2, *N* = 186) = 4.444, *p* = 0.108, gr. 1 vs. gr. 3 *χ*^2^ (2, *N* = 108) = 2.100, *p* = 0.350, gr. 2 vs. gr. 3		
BMI	Cat. 1	11 (4.5%)	7 (2.8%)	2 (0.8%)	16 (6.5%)	4 (1.6%)	7 (2.8%)	8 (3.2%)	5 (2.0%)
	Cat. 2	49 (19.9%)	72 (29.1%)	14 (5.7%)	110 (44.6%)	25 (10.1%)	25 (10.1%)	72 (29.2%)	38 (15.4%)
	Cat. 3	22 (8.9%)	30 (12.1%)	6 (2.4%)	52 (21.1%)	6 (2.4%)	14 (5.7%)	27 (11.0%)	17 (6.9%)
	Cat. 4	15 (6.1%)	18 (7.3%)	1 (0.4%)	30 (12.1%)	4 (1.6%)	6 (2.4%)	17 (6.9%)	11 (4.4%)
Statistical evaluation		*χ*^2^ (6, *N* = 247) = 4.676, *p* = 0.586 *χ*^2^ (2, *N* = 227) = 0.687, *p* = 0.709, normal weight vs. OW + OB			*χ*^2^ (3, *N* = 247) = 2.730, *p* = 0.435 *p* = 0.137, normal weight vs. OW + OB		*χ*^2^ (6, *N* = 247) = 3.817, *p* = 0.702 *χ*^2^ (2, *N* = 227) = 0.709, *p* = 0.701, normal weight vs. OW + OB		
Gender	F	73 (29.6%)	79 (32.0%)	14 (5.7%)	68 (27.5%)	13 (5.3%)	39 (15.8%)	79 (32.0%)	48 (19.4%)
	M	24 (9.7%)	48 (19.4%)	9 (3.6%)	140 (56.7%)	26 (10.5%)	13 (5.3%)	45 (18.2%)	23 (9.3%)
Statistical evaluation		*χ*^2^ (2, *N* = 247) = 4.714, ***p* = 0.095**			*p* = 1.000		*χ*^2^ (2, *N* = 247) = 2.126, *p* = 0.345		
Educational level	Level 1	12 (4.9%)	25 (10.1%)	3 (1.2%)	37 (15.0%)	3 (1.2%)	4 (1.6%)	21 (8.5%)	15 (6.1%)
	Level 2	31 (12.6%)	50 (20.2%)	13 (5.3%)	71 (28.7%)	23 (9.3%)	17 (6.9%)	52 (21.1%)	25 (10.1%)
	Level 3	54 (21.9%)	52 (21.0%)	7 (2.8%)	100 (40.5%)	13 (5.3%)	31 (12.6%)	51 (20.6%)	31 (12.6%)
Statistical evaluation		*χ*^2^ (4, *N* = 247) = 8.945, ***p* = 0.063**, for all study levels *χ*^2^ (2, *N* = 134) = 1.454, *p* = 0.483, level 1 vs. level 2 *χ*^2^ (2, *N* = 153) = 3.839, *p* = 0.147, level 1 vs. level 3 *χ*^2^ (2, *N* = 207) = 6.372, ***p* = 0.041**, level 2 vs. level 3			*χ*^2^ (4, *N* = 247) = 8.952, ***p* = 0.011**, for all groups ***p* = 0.030**, level 1 vs. level 2 *p* = 0.564, level 1 vs. level 3 ***p* = 0.017**, level 2 vs. level 3		*χ*^2^ (4, *N* = 247) = 7.296, *p* = 0.121, for all study levels *χ*^2^ (2, *N* = 134) = 2.329, *p* = 0.312, level 1 vs. level 2 *χ*^2^ (2, *N* = 153) = 5.261, ***p* = 0.072**, level 1 vs. level 3 *χ*^2^ (2, *N* = 207) = 3.017, *p* = 0.221, level 2 vs. level 3		

Values indicated in bold denote statistically significant differences. Q8—Do you read the composition or ingredients list of products before purchasing them?; Q9—Does the information about the composition or ingredients of a product influence your decision to purchase it?; Q10—Do you search for information about additives listed in the ingredients of a product before purchasing it?

**Table 4:** Statistical analysis of water consumption (Q16) of the respondents.

Parameter	Groups	Q16				Statistical Evaluation
		<1 L	1–2 L	2–3 L	>3 L	
Age	18–30 (1)	16 (6.5%)	80 (32.4%)	29 (11.7%)	14 (5.7%)	*χ*^2^ (6, *N* = 247) = 15.949, ***p* = 0.014**, for all age categories *χ*^2^ (3, *N* = 200) = 9.196, ***p* = 0.027**, group 1 vs. group 2 *χ*^2^ (3, *N* = 186) = 8.021, ***p* = 0.046**, group 1 vs. group 3 *χ*^2^ (3, *N* = 108) = 15.949, *p* = 0.232, group 2 vs. group 3
	30–50 (2)	10 (4.0%)	44 (17.8%)	6 (2.4%)	1 (0.4%)	
	50+ (3)	13 (5.3%)	25 (10.1%)	7 (2.8%)	2 (0.8%)	
BMI	Cat. 1	5 (2.0%)	12 (4.9%)	2 (0.8%)	1 (0.4%)	*χ*^2^ (9, *N* = 247) = 18.112, ***p* = 0.034** Normal weight vs. overweight and obese, *χ*^2^ (3, *N* = 227) = 8.643, ***p* = 0.034**
	Cat. 2	23 (9.3%)	87 (35.2%)	20 (8.1%)	5 (2.0%)	
	Cat. 3	7 (2.8%)	32 (13.0%)	15 (6.1%)	4 (1.6%)	
	Cat. 4	4 (1.6%)	18 (7.3%)	5 (2.0%)	7 (2.8%)	
Gender	F	6 (2.4%)	45 (18.2%)	19 (7.7%)	11 (4.4%)	*χ*^2^ (3, *N* = 247) = 16.624, ***p* < 0.001**
	M	33 (13.4%)	104 (42.1%)	23 (9.3%)	6 (2.4%)	
Educational level	Level 1	5 (2.0%)	28 (11.3%)	4 (1.6%)	3 (1.2%)	*χ*^2^ (6, *N* = 247) = 4.033, *p* = 0.672 *χ*^2^ (3, *N* = 153) = 2.100, *p* = 0.552 *χ*^2^ (3, *N* = 153) = 2.647, *p* = 0.449 *χ*^2^ (3, *N* = 207) = 1.542, *p* = 0.672
	Level 2	13 (5.3%)	55 (22.3%)	18 (7.3%)	8 (3.2%)	
	Level 3	21 (8.5%)	66 (26.7%)	20 (8.1%)	6 (2.4%)	

Values indicated in bold denote statistically significant differences. Q16—How much water do you usually consume daily?

**Table 5:** Statistical analysis for Q17 regarding the use of dietary supplements.

Parameter	Groups	Q17		Statistical Evaluation
		Yes	No	
Age	18–30 (1)	83 (33.6%)	56 (22.7%)	*χ*^2^ (2, *N* = 247) = 2.346, *p* = 0.310, for all age categories *p* = 0.215, group 1 vs. group 2 *p* = 0.865, group 1 vs. group 3 *p* = 0.243, group 2 vs. group 3
	30–50 (2)	30 (12.1%)	31 (12.6%)	
	50+ (3)	29 (11.7%)	18 (7.3%)	
BMI	Cat. 1	10 (4.0%)	10 (4.0%)	*χ*^2^ (3, *N* = 247) = 16.856, ***p* < 0.001** Normal weight vs. overweight and obese, ***p* < 0.001**
	Cat. 2	92 (37.3%)	43 (17.4%)	
	Cat. 3	29 (11.8%)	29 (11.8%)	
	Cat. 4	11 (4.4%)	23 (9.3%)	
Gender	F	41 (16.6%)	40 (16.2%)	*p* = 0.134
	M	101 (40.9%)	65 (26.3%)	
Educational level	Level 1	18 (7.3%)	22 (8.9%)	*χ*^2^ (2, *N* = 247) = 4.133, *p* < 0.127 ***p* = 0.056**, level 1 vs. level 2 *p* = 0.268, level 1 vs. level 3 *p* = 0.321, level 2 vs. level 3
	Level 2	60 (24.3%)	34 (13.8%)	
	Level 3	64 (25.9%)	49 (19.8%)	

Q17—Do you use any dietary supplements? Values indicated in bold denote statistically significant differences.

## Data Availability

The original contributions presented in the study are included in the article, further inquiries can be directed to the corresponding author.

## Supplementary Materials

The following supporting information can be downloaded at: https://www.mdpi.com/article/10.3390/nu17050756/s1, Table S1: Statistical analysis of the responses given to Q15 about fats used for cooking.

## Abbreviations

The following abbreviations are used in this manuscript:

DASH	Dietary Approach to Stop Hypertension
EFSA	European Food Safety Authority
FAO	Food and Agriculture Organization of the United Nations
GDPR	General Data Protection Regulation
MVDA	Multivariate Data Analysis
NCD	Non-Communicable Diseases
OPLS-DA	Orthogonal Partial Least Squares Discriminant Analysis
PCA-X	Principal Component Analysis
PUFA	Polyunsaturated Fatty Acids
TWI	Total Water Intake
WHO	World Health Organization

## Appendix A

Questionnaire regarding the dietary habits of the ethnic Hungarian population of Romania

***Personal data***

**I. Age**

a. 18–30

b. 30–50

c. 50+

**II. Gender**

a. Male

b. Female

**III. Last degree completed, or current studies**

a. Secondary education

b. On-going undergraduate studies

c. Undergraduate or graduate degree

**IV. Height (cm)**

_______ (open question)

**V. Weight (kg)**

_______ (open question)

***Dietary patterns***

**Q1. Are you currently following a specific diet?**

a. Yes

*If yes, please specify which diet(s): ______* (open question)

b. No

**Q2. Have you followed a specific diet in the past?**

a. Yes

*If yes, please specify which diet(s): ______* (open question)

b. No

**Q3. Have you ever consulted a dietitian or nutritionist for dietary advice or meal planning?**

a. Yes

b. No

***Purchasing habits***

**Q4. Where do you usually purchase your food? (Select all that apply)**

a. Farmer’s market

b. Local grocery stores

c. Supermarkets

d. Organic grocery stores

**Q5. When purchasing fruits and vegetables, which do you prefer?**

a. Local products (e.g., from nearby farms and markets)

b. Romanian products

c. Imported products

**Q6. When purchasing other groceries (e.g., dairy, meat, packaged goods), which do you prefer?**

a. Local products (e.g., from nearby farms and markets)

b. Romanian products

c. Imported products

**Q7. How do you usually handle your meals? (Select all that apply)**

a. I mostly cook meals at home.

b. I mostly order takeouts or eat at a restaurant.

c. I mostly eat fast food.

***Food composition and labelling***

**Q8. Do you read the composition or ingredients list of products before purchasing them?**

a. Yes, always

b. Occasionally

c. No, never

**Q9. Does the information about the composition or ingredients of a product influence your decision to purchase it?**

a. Yes

b. No

**Q10. Do you search for information about additives listed in the ingredients of a product before purchasing it?**

a. Yes

b. Occasionally

c. No

**Q11. How often do you consume the following types of products? (Frequency question)**

	**Daily**	**Several Times a Week**	**Once a Week**	**Rarely**	**Never**
a. Fruits	☐	☐	☐	☐	☐
b. Vegetables	☐	☐	☐	☐	☐
c. Baked goods (e.g., bread, pastries)	☐	☐	☐	☐	☐
d. Grains (e.g., rice, oats, quinoa)	☐	☐	☐	☐	☐
e. Dairy products (e.g., milk, cheese, yoghurt)	☐	☐	☐	☐	☐
f. Eggs	☐	☐	☐	☐	☐
g. Meats	☐	☐	☐	☐	☐
h. Fish and seafood	☐	☐	☐	☐	☐
i. Processed meats (e.g., salami, sausages, ham)	☐	☐	☐	☐	☐
j. Pasta products	☐	☐	☐	☐	☐
k. Legumes (e.g., beans, lentils, peas)	☐	☐	☐	☐	☐
l. Nuts and seeds (e.g., walnuts, almonds, chia seeds, pumpkin seeds)	☐	☐	☐	☐	☐
m. Animal fats (e.g., butter, lard)	☐	☐	☐	☐	☐
n. Vegetable fats (e.g., sunflower oil, olive oil, coconut oil)	☐	☐	☐	☐	☐
o. Sweets (e.g., chocolate, candies, cake, biscuits)	☐	☐	☐	☐	☐
p. Salty snacks and fast food (e.g., chips, crackers, fries)	☐	☐	☐	☐	☐
q. Sugary soft drinks	☐	☐	☐	☐	☐
r. Alcohol	☐	☐	☐	☐	☐

**Q12. Which of the following protein sources do you use most frequently in your diet? (Select up to three)**

a. Meat and meat products

b. Dairy products

c. Seafood

d. Legumes

e. Seeds

f. Soybeans

**Q13. Which of the following carbohydrate sources do you use most frequently in your diet? (Select up to three)**

a. Baked goods

b. Rice

c. Pasta

d. Potato

e. Sweets and soda

f. Legumes

g. Fruits

h. Vegetables

**Q14. Which of the following lipid sources do you use most frequently in your diet? (Select up to three)**

a. Fruits and vegetables (e.g., avocado, olives, soybeans)

b. Seeds (e.g., walnuts, hazelnuts, chia seeds)

c. Vegetable oils (e.g., sunflower oil, olive oil, coconut oil)

d. Animal fats (e.g., butter, lard)

e. Oily fishes (e.g., salmon, tuna, herring)

**Q15. How often do you use the following types of fats for cooking?**

	**Daily**	**Several Times a Week**	**Once a Week**	**Rarely**	**Never**
a. Butter	☐	☐	☐	☐	☐
b. Sunflower oil	☐	☐	☐	☐	☐
c. Olive oil	☐	☐	☐	☐	☐
d. Other vegetable oils	☐	☐	☐	☐	☐
e. Lards (e.g., pork, duck)	☐	☐	☐	☐	☐

**Q16. How much water do you usually consume daily?**

a. Less than 1 L

b. Between 1–2 L

c. Between 2–3 L

d. Between 3–4 L

e. More than 4 L

**Q17. Do you use any dietary supplements?**

a. Yes

b. No

**Q18. If the answer to the previous question was yes, please specify which types of dietary supplements you use:**

a. Proteins

b. Creatine

c. Vitamins and minerals

d. Immunostimulants

e. Other, please specify _______
