# Supplementary material for: Dietary Habits and Their Correlation with Socio-Demographic Variables Among the Ethnic Hungarian Population of Romania
Source: Nutrients. 2025 Feb 21;17(5):756. doi: 10.3390/nu17050756 (PMC11901452; doi:10.3390/nu17050756)
Supplement: Supplementary file 1 [file nutrients-17-00756-s001.zip › nutrients-3479981-supplementary.pdf]

nutrients MDPI

Supplementary files

Table S1. Statistical analysis of the responses given to Q15 about fats used for cooking.

Parameter	Group	Q15a–Butter			Q15b–Sunflower Oil			Q15c–Olive Oil			Q15d–Other Vegetable Oil			Q15e–Lards		
		**Several Times a Week**	**Occ.**	**Never**	**Several Times a Week**	**Occ.**	**Never**	**Several Times a Week**	**Occ.**	**Never**	**Several Times a Week**	**Occ.**	**Never**	**Several Times a Week**	**Occ.**	**Never**
Age	18–30 (1)	66 (26.7%)	65 (26.3%)	8 (3.2%)	82 (33.2%)	52 (21.0%)	5 (2.0%)	71 (28.7%)	63 (25.5%)	5 (2.0%)	7 (2.8%)	87 (35.2%)	45 (18.2%)	27 (11.0%)	62 (25.1%)	50 (20.3%)
	30–50 (2)	37 (15.0%)	23 (9.3%)	1 (0.4%)	37 (15.0%)	23 (9.3%)	1 (0.4%)	24 (9.7%)	37 (15.0%)	0 (0%)	3 (1.2%)	45 (18.2%)	13 (5.3%)	17 (6.9%)	36 (14.6%)	8 (3.2%)
	50+ (3)	19 (7.7%)	26 (10.5%)	2 (0.8%)	29 (11.7%)	15 (6.1%)	3 (1.2%)	26 (10.5%)	19 (7.7%)	2 (0.8%)	10 (4.0%)	29 (11.8%)	8 (3.2%)	17 (6.9%)	25 (10.1%)	5 (2.0%)
Statistical evaluation		χ^2 (4, N = 247) = 5.863, p = 0.208			χ^2 (4, N = 247) = 2.018, p = 0.732			χ^2 (4, N = 247) = 6.809, p = 0.146			χ^2 (4, N = 247) = 17.359, p = 0.002			χ^2 (4, N = 247) = 19.935, p < 0.001		
BMI	Cat. 1	11 (4.4%)	7 (2.8%)	2 (0.8%)	16 (6.5%)	4 (1.6%)	0 (0%)	12 (4.9%)	7 (2.8%)	1 (0.4%)	0 (0%)	15 (6.1%)	5 (2.0%)	4 (1.6%)	8 (3.2%)	8 (3.2%)
	Cat. 2	74 (30.0%)	56 (22.7%)	5 (2.0%)	82 (33.2%)	48 (19.4%)	5 (2.0%)	67 (27.1%)	65 (26.3%)	3 (1.2%)	9 (3.6%)	89 (36.0%)	37 (15.0%)	37 (15.0%)	60 (24.3%)	38 (15.4%)
	Cat. 3	25 (10.1%)	30 (12.1%)	3 (1.2%)	31 (12.6%)	26 (10.5%)	1 (0.4%)	26 (10.5%)	30 (12.1%)	2 (0.8%)	8 (3.2%)	31 (12.6%)	19 (7.7%)	12 (4.9%)	35 (14.2%)	11 (4.4%)
	Cat. 4	12 (4.9%)	21 (8.5%)	1 (0.4%)	19 (7.7%)	12 (4.9%)	3 (1.2%)	16 (6.5%)	17 (6.9%)	1 (0.4%)	3 (1.2%)	26 (10.5%)	5 (2.0%)	8 (3.2%)	20 (8.1%)	6 (2.4%)
Statistical evaluation		χ^2 (6, N = 247) = 7.920, p = 0.244<br>χ^2 (2, N = 227) = 4.701, p = 0.090 [a]			χ^2 (3, N = 247) = 4.638, p = 0.200<br>χ^2 (2, N = 227) = 0.919, p = 0.632 [a]			χ^2 (6, N = 247) = 2.221, p = 0.898<br>χ^2 (2, N = 227) = 0.500, p = 0.779 [a]			χ^2 (4, N = 247) = 6.874, p = 0.142<br>χ^2 (2, N = 227) = 1.907, p = 0.385 [a]			χ^2 (4, N = 247) = 7.803, p = 0.253<br>χ^2 (2, N = 227) = 5.352, p = 0.069 [a]		
Gender	F	86 (34.8%)	73 (29.6%)	7 (2.8%)	107 (43.3%)	51 (20.6%)	8 (3.2%)	86 (34.8%)	73 (29.6%)	7 (2.8%)	13 (5.3%)	109 (44.1%)	44 (17.8%)	36 (14.6%)	80 (32.4%)	50 (20.2%)
	M	36 (14.6%)	41 (16.6%)	4 (1.6%)	41 (16.6%)	39 (15.8%)	1 (0.4%)	35 (14.2%)	46 (18.6%)	0 (0%)	7 (2.8%)	52 (21.0%)	22 (8.9%)	25 (10.1%)	43 (17.4%)	13 (5.3%)
Statistical evaluation		χ^2 (2, N = 247) = 1.181, p = 0.554			χ^2 (2, N = 247) = 8.197, p = 0.017			χ^2 (1, N = 247) = 1.610, p = 0.204			χ^2 (2, N = 247) = 0.070, p = 0.965			χ^2 (4, N = 247) = 6.344, p = 0.042		
Study level	Level 1	17 (6.9%)	21 (8.5%)	2 (0.8%)	23 (9.3%)	16 (6.5%)	1 (0.4%)	18 (7.3%)	20 (8.1%)	2 (0.8%)	7 (2.8%)	24 (9.7%)	9 (3.6%)	14 (5.7%)	16 (6.5%)	10 (4.0%)
	Level 2	42 (17.0%)	47 (19.0%)	5 (2.0%)	57 (23.1%)	34 (13.8%)	3 (1.2%)	49 (19.8%)	42 (17.0%)	3 (1.2%)	4 (1.6%)	59 (23.9%)	31 (12.6%)	12 (4.9%)	48 (19.4%)	34 (13.7%)
	Level 3	63 (25.5%)	46 (18.6%)	4 (1.6%)	68 (27.5%)	40 (16.2%)	5 (2.0%)	54 (21.9%)	57 (23.1%)	2 (0.8%)	9 (3.6%)	78 (31.6%)	26 (10.5%)	35 (14.2%)	59 (23.9%)	19 (7.7%)
Statistical evaluation		χ^2 (4, N = 247) = 3.506, p = 0.477			χ^2 (4, N = 247) = 0.609, p = 0.962			χ^2 (4, N = 247) = 1.895, p = 0.755			χ^2 (4, N = 247) = 8.816, p = 0.066			χ^2 (4, N = 247) = 17.416, p = 0.002		

[a] comparison of normal weight individuals with overweight and obese respondents. **Q15** - How often do you use the following types of fats for cooking?

 https://doi.org/10.3390/nu17050756
